# Effect of pacing strategy modification on 200 m performance in athletics

**DOI:** 10.3389/fspor.2025.1657245

**Published:** 2025-10-14

**Authors:** Takaya Yoshimoto, Yoshihiro Chiba, Soshi Mizukubo, Kentaro Sato, Hayato Ohnuma, Yohei Takai

**Affiliations:** ^1^ Graduate School of Education, Hyogo University of Teacher Education, Hyogo, Japan; ^2^ Faculty of Management, Josai University, Saitama, Japan; ^3^ Miyazaki Prefectural Sports Association, Miyazaki, Japan; ^4^ Waseda Institute of Sport Sciences, Waseda University, Saitama, Japan; ^5^ Faculty of Health and Welfare, Kobe Women’s University, Hyogo, Japan; ^6^ Faculty of Sports and Life Science, National Institute of Fitness and Sports in Kanoya, Kagoshima, Japan

**Keywords:** split time, pace distribution, race strategy, running speed, step frequency, step length, re-acceleration

## Abstract

In the 200 m sprint, it remains unclear whether modifying pacing distribution can lead to improved performance. This study aimed to address this question through three approaches: (1) cross-sectional analysis of world-class sprinters, (2) longitudinal analysis of an elite Japanese sprinter, and (3) an intervention involving pacing strategy modification for that sprinter. The study comprised three components: (1) cross- sectional analysis of 53 official races by world-class sprinters, (2) longitudinal analysis of 8 official races by an elite Japanese sprinter, and (3) a pacing intervention based on these analyses. Pacing distribution was assessed using two indices: the percentage of each 10 m split time relative to the 200 m record (%ST), and the percentage of each 100 m split time relative to the 100 m personal record (%PR). Cross- sectional analysis showed that world-class sprinters tended to sprint relatively slow in the first half and faster in the second half of the 200 m. Longitudinal analysis indicated that the Japanese sprinter achieved faster overall times when his speed in the 100–200 m segment was higher. Based on these findings, the modified pacing strategy improved his 200 m record from 20.43 to 20.14 s. These findings suggest that a pacing strategy focusing on maintaining speed in the latter half of the race, by moderating early acceleration, may contribute to performance improvement in elite-level 200 m sprinting.

## 1 Introduction

The 200 m race in athletics is a sprinting event where athletes transition from a curved to a straight track at extremely high speeds (1–4). Sprinters typically complete the distance in approximately 20 s (3, 4). In simulated 200 m events, anaerobic energy supply declines significantly within that 20 s duration (5). A previous study has demonstrated that anaerobic energy stores are substantially depleted during the 200 m sprint (6). This is likely due to rapid declines in ATP-PCr levels and muscle glycogen at the onset of high-intensity exercise (7–9). Therefore, 200 m performance appears to depend on the magnitude of anaerobic work capacity.

The 200 m race differs from the 100 m in that sprinters must transition from a curve to a straight. Similar to the 400 m, the 200 m also begins on a curve, but sprinters are required to achieve high running speeds primarily through greater step frequency (10–12). An Olympic 200 m gold medalist not only attains a high running speed but also maintains a high step frequency in the latter half of the race, facilitated by short ground contact times (13). It remains unclear whether this strategy enables maximal speed in the first 100 m that is then sustained or whether athletes deliberately moderate early acceleration. Little information is available on pacing strategies in 200 m track events. In 400 m races, an initial high step frequency allows sprinters to reach high speed in the first 200 m. However, this often leads to a rapid decrease in frequency and a significant speed drop in the second 200 m (14–16). Conversely, controlling step frequency in the first half and moderating speed minimizes the frequency in the second half, helping to maintain high speed throughout (14, 15). In cyclic knee-joint movement, decreases in external force and mechanical power are less pronounced at lower tempos than in higher tempos (17). Additionally, in 400 m races, improved performance is associated with increased stride length (15). In 400 m races, adopting a more driven pacing strategy (around 93% of the 200 m personal best) has been linked to a smaller decline in both step frequency and step length during the latter half of the 400 m race, together with a reduced discrepancy between the first and second halves compared with a more leading strategy (98%) (12). Additionally, personal record improvement is associated with increased stride length with more or less constant step frequency (15). Based on the aforementioned studies, we hypothesized that moderating step frequency in the first half of the race, thereby suppressing speed acquisition, could reduce the subsequent frequency decline, ultimately allowing athletes to maintain high speed and long strides.

Therefore, this study aimed to investigate the relationship between 200 m performance and pace distribution in elite sprinters and to clarify an effect of pacing modification on race outcomes and spatiotemporal parameters. To test this, we analyzed 200 m races to clarify the relationship between 200 m records and pace distribution across the world's elite sprinters and within an elite sprinter. Furthermore, we examined how modifying the pacing strategy of the Japanese sprinter affected his 200 m record and spatiotemporal parameters.

## 2 Materials & methods

### 2.1 Data collection and procedures

To test the aforementioned hypothesis, we analyzed 53 official 200 m races to elucidate the characteristics of pacing strategy employed by world male elite sprinters competing in the Olympics and Diamond League series (Experiment 1; Exp. 1) (18, 19). In total, 207 races were initially collected; however, when sprinters appeared in multiple races, only their best-performance race was included in the analysis. The tracking data were obtained from the official “Race Analysis Graphical” reports provided by OMEGA Timing, the official timekeeper of the Diamond League and the Olympic Games. For the 2023 season, these data were based on OMEGA's Real-Time Tracking System, which utilizes a lightweight chip embedded in the bib number worn on the athletes’ chests. The system transmits real-time data to 16 receivers positioned around the outside of the track and 4 on the inside, capturing split times and speed data in real time with a resolution of 0.01 s (i.e., to the hundredth of a second). Official finishing times were determined by the Scan’O’Vision Myria photofinish camera, which records up to 10,000 digital images per second (20, 21). For the 2024 season, including the Paris Olympic Games, OMEGA introduced a new system featuring Computer Vision technology for tag-free athlete tracking and the Scan’O’Vision Ultimate capable of capturing up to 40,000 images per second (22, 23). Although we did not collect the data ourselves, the data source is internationally recognized and serves as the official basis for performance data at the Diamond League and Olympic Games, which guarantees the highest standards of validity and reliability for cross-sectional analyses. Additionally, we analyzed eight races of a Japanese elite sprinter, including data on 200 m times and split times for each 100 m segment (Exp. 2). Combining the results of Exp. 1 and Exp. 2, we created a training program to modify the sprinter's pace-distribution strategy. We then prescribed this program to the elite Japanese sprinter and examined whether altering his pacing strategy would change his pace distribution and yield a new 200 m race record (Exp. 3). This study was approved by the ethics committee of the National Institute of Fitness and Sports in Kanoya (Approval Number: 23-1-39), and all procedures conformed to the Declaration of Helsinki. Prior to participation, the athlete was fully informed of the study's purpose and risks and provided written consent.

### 2.2 Study design in Exp. 1

Fifty-three races by 53 sprinters who competed in the Diamond League series during 2023 and 2024, as well as in the 2024 Paris Olympics (18, 19), were analyzed. The data included their 200 m times ranging from 19.47 s to 20.87 s, broken down into 10 m segments, along with their 100 m personal records. For sprinters who competed in multiple races, only their fastest time was included in the analysis. We categorized the races into two groups: those completed under 20 s (fast races, *N* = 9) and those completed in over 20 s (slow races, *N* = 44). This threshold was adopted based on the historical observation that no athlete has failed to reach the World Championships final with sub-20 s performance in the 200 m (24). Therefore, a time under 20 s was considered a practical indicator of world-class performance. To clarify the pacing distribution associated with faster 200 m records, we computed the following variables.

#### 3.2.1 Running speed per 10 m and percentage of each 10 m split time relative to the 200 m record (%St)

Running speed per 10 m was derived from the 10 m split time (25). The %ST was calculated by dividing the time for each 10 m split by the 200 m record time and expressing it as a percentage. A higher %ST value indicates that sprinters ran relatively slower during that specific segment compared to other segments of the races.

#### 3.2.2 Percentage of passing time at each 100 m segment of a 200 m race relative to 100 m personal record (%PR)

The %PR for each 100 m segment was calculated by dividing the time for that segment by the sprinter's personal best time for 100 m and expressing it as a percentage (25). A higher %PR value indicates that sprinters ran at a speed closer to their 100 m personal record. The individual 100 m records used in the analysis were obtained from the same year as the corresponding 200 m race records. The analysis of %PR was based on 46 races, as seven sprinters did not have a 100 m record in the same season (faster races, *N* = 8; slower races, *N* = 38).

### 2.3 Study design in Exp. 2

To validate the effectiveness of the pacing strategy proposed in Exp. 1, an elite Japanese sprinter (MS) (age 25 years, height 1.736 m, body mass 72.0 kg), who had a PR of 10.14 s for 100 m and 20.43 s for 200 m prior to modifying his pacing strategy, implemented this strategy during an official national race. MS also served as a reserve member of the 4 × 100 m relay team at the 2023 World Athletics Championships. MS engaged in specific training program for at least 3 h per day, 5 days per week, under the guidance of coaches YC and TY. His 200 m race time was recorded at 20.43 s during the 65th East Japan Corporate Athletics Championship. MS initially adopted a pacing strategy that emphasized sprinting as fast as possible from the start. In addition to the results of Exp. 1, the data from before the MS changed their pace distribution was also examined, and based on those results, the MS adjusted their pace-distribution strategy with the advice of the coaches.

To determine the pacing strategy before modifying his race strategy, we analyzed the data (*N* = 8) from MS's 200 m race using the methods mentioned in previous studies (11, 26, 27). MS participated in the 6th Fuji North Foot World Trials held on August 18, 2024. MS's races were recorded using a digital video camera (Lumix FZ300, Panasonic, Japan; frame rate: 239.76 fps; shutter speed: auto; f-ratio: 2.8; resolution: 640 × 480 pixels) for two races and obtained from internet broadcasts for seven races. Running time, speed, and spatiotemporal variables were analyzed at each 100 m segment (Table 1). The camera was positioned in the spectator stands, directly aligned with the finish line on the back straight and elevated above track level. This placement enabled clear lateral capture of both the 100 m mark and the finish line. To account for the timing discrepancies characteristic of broadcast footage, the analysis was performed based on a frame rate of 29.97 fps without relying on the on-screen timer. The start time in the 200 m race was estimated by back-calculating from the official finish time and the frame number at which MS crossed the finish line, taking into account the video frame rate.

**Table 1:** Correlation coefficients between the 200 m race records before pace modification and the measured variables for MS.

Variable	Segment	Correlation with 200 m race record
Running speed	0–100 m	−0.302
	100–200 m	−0.965*
Step frequency	0–100 m	−0.387
	100–200 m	−0.646
Step length	0–100 m	0.402
	100–200 m	−0.090
%ST	0–100 m	−0.866*
	100–200 m	0.866*
%PR	0–100 m	0.369
	100–200 m	−0.905*

%ST, the percentage of each 100 m segment time relative to the 200 m race time; %PR, the percentage of each 100 m segment time relative to the personal record of the 100 m race.

* Denotes *p* < 0.05.

If a line was present at the 100 m mark, the time to reach that mark was measured directly. If no line was visible at the 100 m mark, we performed a linear regression using the split times at the entrance (80 m mark) and exit (110 m mark) of the fourth-corner takeover zone to interpolate the 100 m time. We then calculated the average running speed for each segment by dividing segment distance by its time. Step frequency was obtained by counting ground contacts within each 100 m segment and dividing that count by the segment time. Finally, we calculated average step length for each segment by dividing average running speed by average step frequency.

### 2.4 Concept for the proposed race strategy and sprint training program (Exp. 3)

Based on the pace distributions obtained in Exp. 1 and Exp. 2, MS adjusted his race strategy to run relatively slower in the first half and faster in the second half than in his previous races. Target values were set at 96% (10.58 s) for the 0–100 m segment and 106% (9.58 s) for the 100–200 m segment, relative to his personal best 100 m record.

In addition to regular training—warm-up exercises (e.g., medicine-ball throws, mini-hurdle high knees, hurdle drills), resistance training (e.g., squats, deadlifts, power cleans, bench presses), and plyometric drills (e.g., hurdle jumps, bounding, hopping)—he performed sprint sessions focused on pacing twice weekly from July 1 to August 17, 2024. The sprint program comprised distances of 120, 150, 200, and 250 m. In each drill, MS completed the first 100 m in no less than 10.60 s, regardless of total distance, then sprinted maximally over the final 100 m. For 200 m repetitions, he was further instructed to control speed up to the 80 m mark, re-accelerate between 80 m and 110 m (exit of the curve), and sprint the last 100 m at maximal effort. Other training loads during the intervention were kept consistent with previous levels.

During this intervention period, no races were run using the pre-intervention pacing strategy. We evaluated its effectiveness by comparing post-intervention race records and spatiotemporal variables with those from previous official races. To ensure adherence to the desired pacing strategy, split-time targets were provided during training, and real-time verbal feedback reinforced appropriate pacing.

### 2.5 Statistical analyses

Descriptive statistics are presented as means and standard deviations (SDs). All analyses were conducted using SPSS software (version 26.0; IBM, Armonk, NY, USA).

In Exp. 1, a two-way repeated-measures analysis of variance was conducted to assess main effects and interactions for running speed, %ST in each 10 m segment, and %PR in each 100 m segment between faster and slower races. When interactions were significant, simple main-effects tests were carried out for *post hoc* comparisons. Additionally, Pearson's correlation coefficient (r) was used to examine the relationship between 200 m race time and each independent variable.

In Exp. 2, Pearson's product-moment correlation coefficients (r) were calculated between 200 m race times and running speed for each 100 m segment, step frequency, step length, %ST, and %PR across the eight races MS completed prior to the pacing trial.

In Exp. 3, small-worth criteria (SWCs) were established based on the mean and SD of each 100 m split time from those eight pre-intervention races, with each SD multiplied by 0.2 to derive the SWC (28). We then determined whether each independent variable deviated from its SWC following the pace-distribution modification.

## 3 Results

### 3.1 Comparisons of faster races to slower races in the independent variables during the 200 m races (Exp. 1)

**Figure 1:**
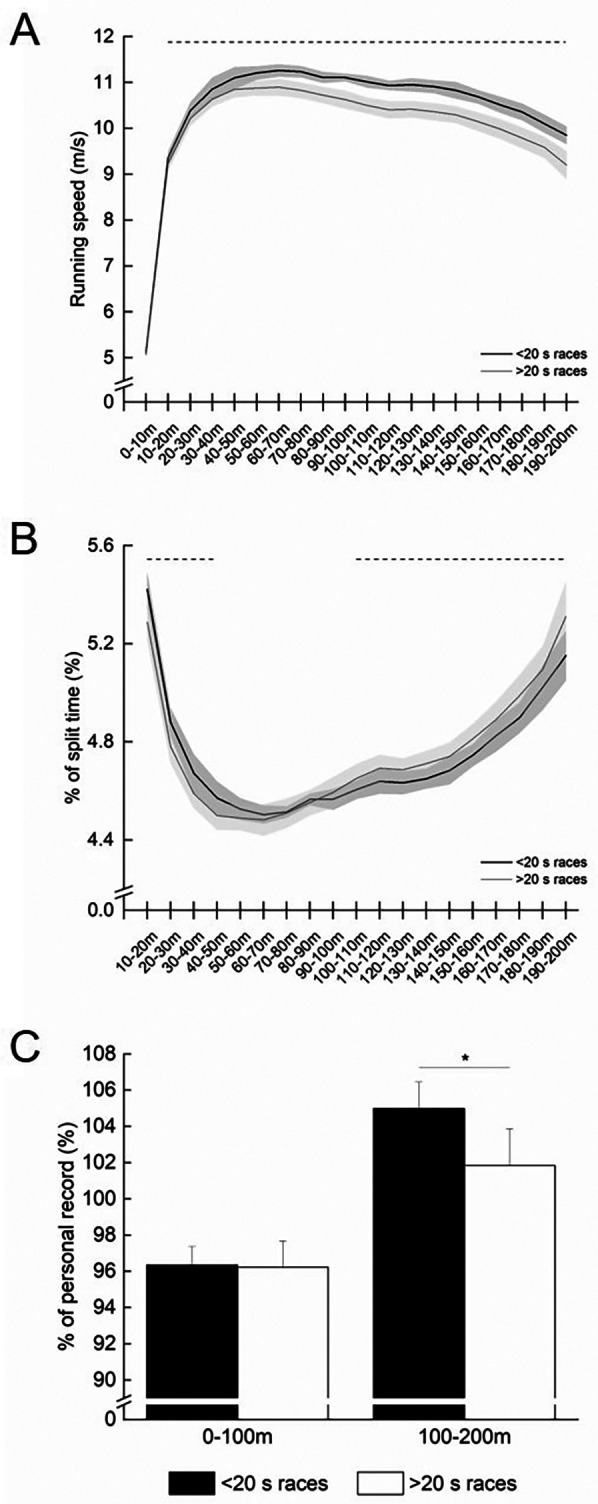
**(A)** running speed for each 10 m segment during 200 m races. **(B)**The percentage of each 10-m segment time relative to the 200 m race time (%ST). The black solid line represents data for faster races (<20 s), while the grey solid line represents data for slower races (>20 s). The dotted line indicates segments where significant group-related differences are found (*p* < 0.05). The %ST in the 0–10 m segment is not shown for readability due to its higher value. In the corresponding section, faster races exhibited a significant higher %ST compared to slower races (29.47% vs. 28.79%). **(C)** Comparison of the percentage of each 100 m segment time relative to the personal record of the 100-m race (%PR) between the 0–100 and 100–200 m sections. The black filled bar represents faster races, and the grey filled bar represents slower races. * denotes a significant difference (*p* < 0.05).

Faster races exhibited higher running speeds across almost all segments compared with slower races, except the 0–10 m segment (Figure 1A). The %ST values for the first 50 m were greater in faster races; conversely, %ST values from 100 m to 200 m were smaller in faster races (Figure 1B). Additionally, %PR in the 100–200 m segment was higher in faster races, whereas no significant difference was observed in %PR for the 0–100 m segment (Figure 1C).

### 3.2 Associations of 200 m times with the independent variables across all races (Exp. 1)

**Figure 2:**
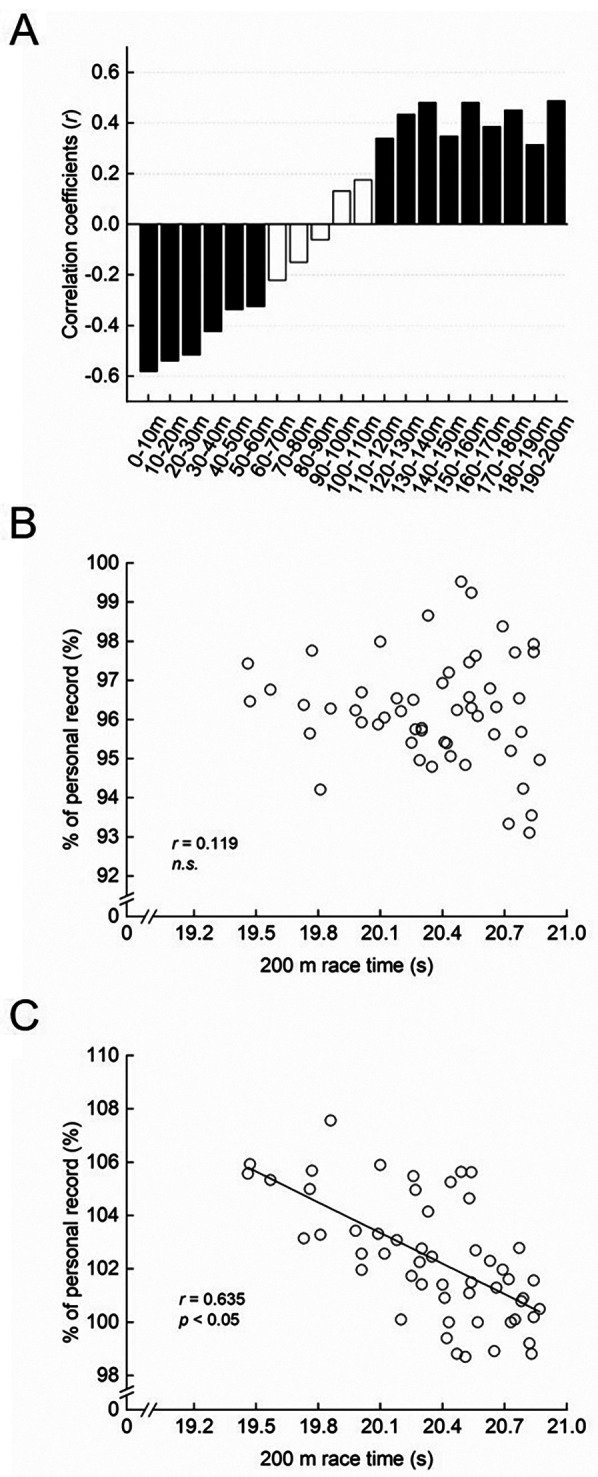
**(A)** correlation coefficients between the 200 m race record and the percentage of each 100-m segment time relative to the 200 m race time (%ST) for each 10 m section. The black filled bars indicate significant correlations (*p* < 0.05), while the white filled bars indicate non-significant correlation. **(B)** Relationship between the 200 m race record and the percentage of each 100 m segment time relative to the personal record of the 100 m race (%PR) in the first half of a race. **(C)** Relationship between the 200 m race record and %PR in the second half of a race.

Across all races (Exp. 1), almost all 10 m segment mean running speeds and maximal running speed were negatively correlated with 200 m times, except in the 0–10 m segment. In contrast, the 200 m time showed significant negative correlations with %ST in the first 70 m and significant positive correlations with %ST from 110 m to 200 m (Figure 2A).

The %PR in the second half of the 200 m was significantly negatively correlated with overall 200 m time (Figure 2B), whereas no significant correlation was found for %PR in the first half (Figure 2C).

### 3.3 Effects of changing pace distribution during a 200 m race on the independent variables (Exp. 2 and 3)

Table 1 shows the relationship between MS's pre-intervention 200 m race record and running speed, step frequency, step length, %ST, and %PR for each 100 m segment. A better 200 m performance was associated with higher values in the 100–200 m segment. After pace-specific sprint training, MS improved his personal best from 20.43 s to 20.14 s, a reduction of 0.29 s. Running speed in the first 100 m remained below the SWC threshold, whereas speed in the second 100 m exceeded it (Table 2). Similarly, step frequency and step length in the second 100 m surpassed SWC thresholds, while both variables in the first 100 m stayed within the SWC range (Table 2).

**Table 2:** 100 m race season record, 200 m race time, segment time, running speed, step frequency, step length and data collection method in MS.

Date	Season record [s]		Time [s]		Speed [m/s]		Frequency [Hz]		Length [m]		Method of data collection
	100 m	200 m	0–100 m	100–200 m	0–100 m	100–200 m	0–100 m	100–200 m	0–100 m	100–200 m	
2020.9.13	10.14	20.75	10.55	10.20	9.48	9.80	4.62	4.72	2.05	2.08	Internet broadcasts
2021.3.28	10.33	20.92	10.49	10.43	9.53	9.59	4.65	4.55	2.05	2.11	Internet broadcasts
2022.4.10	10.27	20.94	10.57	10.37	9.46	9.64	4.63	4.54	2.04	2.12	Internet broadcasts
2023.5.21	10.20	20.43	10.47	9.96	9.55	10.04	4.79	4.74	1.99	2.12	Internet broadcasts
2023.6.3	10.20	20.66	10.48	10.18	9.54	9.82	4.84	4.52	1.97	2.17	Internet broadcasts
2023.7.28	10.20	20.55	10.59	9.96	9.44	10.04	4.55	4.84	2.07	2.07	Internet broadcasts
2024.6.27	10.15	20.55	10.47	10.08	9.55	9.92	4.76	4.65	2.01	2.14	Digital video camera
2024.6.28	10.15	20.61	10.53	10.08	9.50	9.92	4.68	4.65	2.03	2.14	Digital video camera
Mean	10.21	20.68	10.52	10.16	9.51	9.85	4.69	4.65	2.03	2.12	–
SD	0.06	0.18	0.05	0.17	0.04	0.17	0.10	0.11	0.03	0.03	–
SWC	10.19–10.22	20.64–20.71	10.51–10.53	10.12–10.19	9.50–9.52	9.81–9.88	4.67–4.71	4.63–4.67	2.02–2.03	2.11–2.12	–
2024.8.18	**10** **.** **15**	**20**.**14**	**10**.**60**	**9**.**54**	**9**.**43**	**10**.**48**	4.68	**4**.**86**	2.02	**2**.**16**	Internet broadcasts
Feasible	**Favor**	**Favor**	**Favor**	**Favor**	**Favor**	**Favor**	Disfavor	**Favor**	Disfavor	**Favor**	–

Bold values indicate results outside the SWC range.

%ST increased in the first 100 m segment but decreased in the second, relative to the SWC threshold (Table 3). Conversely, %PR decreased in the first 10 m segment but increased in the second, compared with the SWC threshold (Table 2).

**Table 3:** Percentage of each 100 m segment 200 m race, percentage of time of each 100 m segment relative to personal record of 100 m race in MS.

Date	%ST [%]		%PR [%]	
	0–100 m	100–200 m	0–100 m	100–200 m
2020.9.13	50.84	49.16	96.11	99.41
2021.3.28	50.14	49.86	98.47	99.04
2022.4.10	50.48	49.52	97.16	99.04
2023.5.21	51.25	48.75	97.42	102.41
2023.6.3	50.73	49.27	97.33	100.20
2023.7.28	51.53	48.47	96.32	102.41
2024.6.27	50.95	49.05	96.94	100.69
2024.6.28	51.09	48.91	96.39	100.69
Mean	50.88	49.12	97.02	100.49
SD	0.44	0.44	0.15	0.27
SWC	50.79–50.96	49.04–49.21	96.87–97.17	100.21–100.76
2024.8.18	**52**.**63**	**47**.**37**	**95**.**75**	**106**.**39**
Feasible	**Favor**	**Favor**	**Favor**	**Favor**

%ST, the percentage of each 100 m segment time relative to the 200 m race time; %PR, the percentage of each 100 m segment time relative to the personal record of the 100 m race.

Bold values indicate results outside the SWC range.

## 4 Discussion

In this study, to examine pace distribution, we analyzed the percentage of each segment within the 200 m race (%ST) and the percentage of each segment relative to each sprinter's 100 m personal best time (%PR). Results from Exp. 1 showed that 200 m races completed in under 20 s exhibited smaller %ST and higher %PR in the second half than races lasting 20 s or more. Exp. 2 revealed that better 200 m records correlated with higher performance in the 100–200 m segment of MS's previous races. The pacing strategy derived from these observations successfully improved an elite Japanese sprinter's 200 m time in Exp. 3. Additionally, analysis of MS's pacing in his record race showed that, in the first 100 m, his running speed and %PR fell below SWC values derived from pre-intervention races while %ST was higher, indicating more controlled early pacing. In the 100–200 m segment, his running speed, step frequency, step length, and %PR all exceeded SWC thresholds, whereas %ST was lower. These findings demonstrate that MS successfully executed the targeted pace distribution, optimizing his second-half performance. Therefore, modifying strategy from emphasizing early acceleration to prioritizing sustained speed in the second half may enhance personal records in the 200 m.

This is the first study to characterize pace development among elite 200 m sprinters and to demonstrate that adjusting strategy based on these characteristics can enhance performance. Notably, MS maintained his usual training regimen throughout the observation period, with the sole addition of pacing strategy drills, and no significant improvements occurred in manually recorded sprint times during standard sessions. As Tables 2, 3 show, pacing modifications were clearly reflected in race data. For example, although MS recorded his slowest 0–100 m split (10.60 s) post-intervention—slower than his slowest pre-intervention split (10.57 s; finish time: 20.94 s)—he achieved a personal best 200 m time of 20.14 s thanks to a fastest-ever 100–200 m split (9.54 s). Based on Exp. 1, we adjusted pace distribution for MS in a 200 m race. Consequently, he improved his personal best by 0.29 s, from 20.43 s to 20.14 s, surpassing the 20.16 s qualifying standard for the 2025 World Championships in Tokyo. This performance also exceeds the 20.31 s mark required to reach the final of the 2024 Paris Olympics, matching the 7th-place finalist's time. These findings indicate that performance gains stemmed from the revised pacing strategy rather than general training adaptations.

While it is widely recognized that the first half of a 200 m sprint is naturally slower due to the block start, curve running, and early acceleration (29), our study quantifies how performance in the latter half distinguishes sub-20 s outcomes. Although Exp. 3 represents a single-case intervention, it rests on robust evidence from 207 race observations of 53 elite male sprinters (Exp. 1). By combining cross-sectional analysis with a longitudinal case study and implementing a targeted pacing intervention, we demonstrate both the practicality and efficacy of this approach. These insights may guide individualized coaching strategies, even if not universally applicable.

In this study, 200 m races completed in under 20 s displayed consistently higher running speeds throughout than those lasting 20 s or longer. Moreover, maximal running speed was strongly and significantly correlated with 200 m performance for all segments except the 0–10 m segment. In 100 m events, elite sprinters attain higher maximal speeds than average sprinters, with differences becoming particularly pronounced after the 30 m mark (30). As in the 100 m sprint, the 200 m event underscores that achieving high running speed is crucial for superior performance.

The pace distribution analysis showed that elite 200 m sprinters exhibited a larger %ST in the 0–60 m segments and a smaller %ST in the 110–200 m segments than those exhibited by sub-elite sprinters, suggesting a strategy of slower pacing in the first half and faster pacing in the second. Furthermore, elite sprinters' %PR was higher in the 100–200 m segment than that of sub-elite athletes.

When examining the race after the pacing strategy modification, the %PR in the 0–100 m segment was 95.75%, and that in the 100–200 m segment was 106.39%, both falling outside the SWC range calculated from MS's previous race data. This suggests that in order to maximize running speed in the 100–200 m segment, MS could control running speed in the 0–100 m segment. At both the 2023 World Championships and the 2024 Paris Olympics, analysis of the 200 m winners showed a renewed increase in running speed between the 110–120 m and 120–130 m segments (Supplementary Data S1), a phenomenon hereafter referred to as re-acceleration. Similarly, the Biomechanical Report of the 2017 IAAF World Championships documented a speed-maintenance strategy with re-acceleration in those same segments (31). In this study, %PR in the second half of the 200 m was significantly negatively correlated with race time. These findings indicate that elite 200 m sprinters strategically manage speed and adopt a pace distribution, including re-acceleration when transitioning from the curve to the straight, that enables higher second-half speeds. A possible factor enabling re-acceleration in the latter half of the race may be due to changes in the recruitment patterns of the ATP-PCr, glycolytic, and oxidative energy systems (12, 32, 33). In maximal sprinting, the ATP-PCr system, which provides the highest power output (33), is rapidly depleted, after which the relative contributions of glycolysis and oxidative phosphorylation increase (6, 32). Therefore, in the 200 m sprint, starting at full intensity leads to a rapid depletion of ATP-PCr stores, whereas moderating early speed can attenuate ATP-PCr utilization, thereby preserving energy supply for speed maintenance and re-acceleration in the latter half of the race. Furthermore, in the 400 m sprint, it has been demonstrated that when sprinters pass the 200 m point at 93% of their personal record for 200 m, compared with passing at 98%, the 0–200 m split time and step frequency in the first 200 m were significantly lower, while both blood lactate concentration at the finish and 400 m race time showed large effect sizes, indicating a tendency toward lower values in the 93% condition (12). This finding suggests that in the 400 m sprint, adjusting step frequency can be used to control early race speed, thereby suppressing glycolytic energy utilization in the first half of the race and preserving glycolytic energy supply for speed maintenance and re-acceleration in the latter stages. In the present study, although step frequency in the 0–100 m segment of the 200 m sprint was within the SWC range, running speed was outside the SWC range, tending to be lower than in previous races. Therefore, adjusting running speed in the early phase might have suppressed glycolytic energy consumption, thereby contributing to re-acceleration passing at 100 m.

In the current study, although step frequency and step length in the 0–100 m segment remained within the SWC, running speed fell below this threshold, suggesting a deliberate pacing adjustment in the early phase. In contrast, a previous study of a 400 m sprinter (15) showed that shifting from a front-loaded strategy to a more conservative first half enabled the athlete to lower his personal best from 45.31 s to 44.77 s. That improvement was attributed to limiting early step frequency while maintaining long step length throughout. Unlike the 400 m approach, where only step frequency was reduced, our findings indicate that both step frequency and step length were intentionally curtailed in the first half of the 200 m to decrease early running speed.

## 5 Limitation

Some limitations warrant consideration in this study. First, the pacing intervention involved a single elite sprinter, and video analysis was confined to 100 m segments because the footage was recorded at 29.97 fps, which corresponds to 0.033 s intervals and may introduce a potential error of approximately 0.01–0.02 s compared with official records measured to 0.01 s. Future studies should recruit large samples and use high-frame-rate recordings with more fine-grained segmental analyses to test generalizability.

Second, the intervention lacked a control trial in which the athlete replicated the race under matched conditions using the previous pacing strategy. Consequently, evaluation relied on comparisons with the athlete's past official records. Although target split times and real-time verbal feedback reinforced adherence to the new strategy, the absence of a repeated race under the former strategy limits isolation of intervention effects from physiological or contextual factors.

Third, this study examined pace distribution changes only in running speed and spatiotemporal variables—step frequency and step length—but pacing modifications may also affect kinematics and physiological responses. Therefore, testing this strategy in sprinters with varied performance levels and characteristics is necessary.

Fourth, environmental factors such as wind and track conditions, as well as the competitive level of each sprinter, may also influence pacing outcomes, and future studies should consider these aspects.

Finally, because this study focused on male sprinters, future research should include female athletes. Addressing these limitations will strengthen subsequent investigations.

## 6 Practical applications

For sprinters such as MS, who initiate the race with maximal effort, adjusting both step frequency and step length to achieve approximately 96% of their 100 m personal best during the first 100 m may help optimize performance. In training, sprinters may target no more than 96% of their 100 m personal best in the first 100 m and then emphasize attaining and maintaining near-peak speed in the latter straight. Furthermore, sprinters already adopting this type of pace distribution could further improve their 200 m performance by lowering their 100 m personal best. These insights emphasize the importance of tailoring pacing strategies to individual characteristics when seeking to maximize 200 m sprint performance. In practice, coaches may monitor such pacing strategies by using timed training segments and providing sprinters with real-time feedback on split times to reinforce the desired distribution.

## 7 Conclusion

In this study, we investigated the relationship between 200 m race times and pacing strategies among world-class sprinters and an elite Japanese sprinter. Inter-subject analysis showed that world-class athletes tend to run relatively slower in the first half and faster in the second half. A within-subject analysis of the Japanese sprinter revealed that higher running speed in the 100–200 m segment was associated with faster 200 m times across eight races. Guided by these findings, we modified his pacing strategy to emphasize latter-half speed, improving his personal best from 20.43 s to 20.14 s. These results suggest that moderating early-phase acceleration to sustain speed in the latter half may enhance performance in elite 200 m sprinters.

## Data Availability

The original contributions presented in the study are included in the article/Supplementary Material, further inquiries can be directed to the corresponding authors.
