# Supplementary figures and images for: Effect of pacing strategy modification on 200 m performance in athletics
Source: Front Sports Act Living. 2025 Oct 14;7:1657245. doi: 10.3389/fspor.2025.1657245 (PMC12558870; doi:10.3389/fspor.2025.1657245)

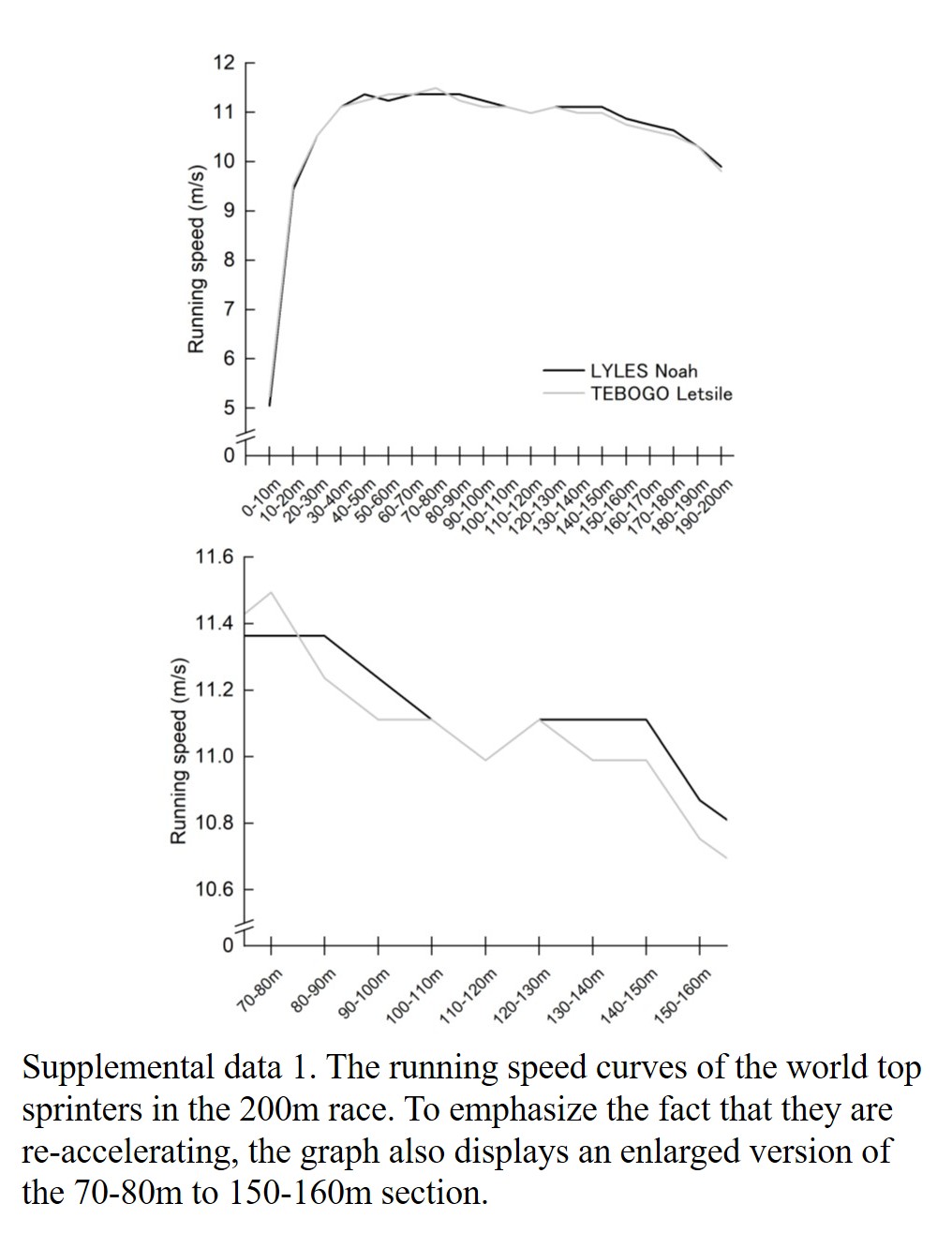

Supplement: Supplementary file 1 [file Image1.jpeg]
